# Information Sharing Practices Between US Hospitals and Skilled Nursing Facilities to Support Care Transitions

**DOI:** 10.1001/jamanetworkopen.2020.33980

**Published:** 2021-01-14

**Authors:** Julia Adler-Milstein, Katherine Raphael, Terrence A. O’Malley, Dori A. Cross

**Affiliations:** 1 Department of Medicine, Center for Clinical Informatics and Improvement Research, University of California, San Francisco, San Francisco; 2 Department of Health Policy, Harvard T.H. Chan School of Public Health, Boston, Massachusetts; 3 Department of Medicine, Massachusetts General Hospital, Boston; 4 Department of Health Policy and Management, University of Minnesota School of Public Health, Minneapolis

## Abstract

**Importance:**

Patient transitions from hospitals to skilled nursing facilities (SNFs) require robust information sharing. After a decade of investment in health information technology infrastructure and new incentives to promote hospital-SNF coordination in the US, the current state of information sharing at this critical transition is unknown.

**Objective:**

To measure the completeness, timeliness, and usability of information shared by hospitals when discharging patients to SNFs, and to identify relational and structural characteristics associated with better hospital-SNF information sharing.

**Design, Setting, and Participants:**

Survey of 500 SNFs from a US nationally representative sample (265 respondents representing 471 hospital-SNF pairs; response rate of 53.0%) that collected detailed data on information sharing that supports care transitions from each of the 2 hospitals from which they receive the largest volume of patient referrals. Survey administration occurred between January 2019 and March 2020.

**Main Outcomes and Measures:**

Overall assessment of information completeness, timeliness, and usability using 5-point Likert scales. Detailed measures, including (1) completeness—routine sharing of 23 specific information types; (2) timeliness—how often information arrived after the patient; and (3) usability—whether information was duplicative, extraneous, or not tailored to SNF needs. In addition, 8 relational characteristics (eg, shared staffing, collaborative meetings, and referral volume) and 10 structural characteristics (eg, size, ownership, and staffing) were assessed as potential factors associated with better information sharing.

**Results:**

Of 471 hospital-SNF pairs, 64 (13.5%) reported excellent performance on all 3 dimensions of information sharing, whereas 141 (30.0%) were at or below the mean performance on all dimensions. Social status (missing in 309 pairs [65.7%]) and behavioral status (missing in 319 pairs [67.7%]) were the most common types of missing information. Receipt of hospital information was delayed, sometimes (159 pairs [33.8%]) or often (77 pairs [16.4%]) arriving after the patient. In total, 358 pairs [76.0%] reported at least 1 usability shortcoming. Having a hospital clinician on site at the SNF was associated in multivariate analysis with more complete (odds ratio, 1.72; 95% CI, 1.07-2.78; *P* = .03), timely (odds ratio, 1.76; 95% CI, 1.08-2.88; *P* = .02), and usable (odds ratio, 1.64; 95% CI, 1.02-2.63; *P* = .04) information sharing. Hospital accountable care organization participation was associated with more timely information sharing (odds ratio, 1.88; 95% CI, 1.13-3.14; *P* = .02).

**Conclusions and Relevance:**

In this study, US SNFs reported significant shortcomings in the completeness, timeliness, and usability of information provided by hospitals to support patient transitions. These shortcomings are likely associated with a suboptimal transition experience. Shared clinicians represent a potential strategy to improve information sharing but are costly. New payment models such as accountable care organizations may offer a more scalable approach but were only associated with more timely sharing.

## Key Points

**Question:**

What is the current state of information sharing to support care transitions between hospitals and skilled nursing facilities (SNFs) in the US, and what characteristics are associated with better sharing?

**Findings:**

In a US nationally representative survey that included responses from 471 hospital-SNF pairs about information sharing, SNFs reported that key information was often missing (functional, mental, and behavioral status as well as whom to contact at the hospital with follow-up questions), delayed (often arriving after the patient), and difficult to use (discharge documents with duplicative and extraneous information). Having a hospital clinician on site at the SNF was associated with more complete, timely, and usable information sharing.

**Meaning:**

This study finds shortcomings across numerous dimensions of information sharing, raising concerns about patients’ transitional care experience from hospitals to SNFs.

## Teaser

This survey study evaluates the current state of the completeness, timeliness, and usability of information sharing, and identifies characteristics associated with better sharing, to support patient care transitions between hospitals and skilled nursing facilities.

## Introduction

Patients transitioning between care settings experience substantial disruptions in continuity that affect the quality and safety of their care.^1,2,3^ Poor information sharing at the time of hospital discharge contributes to this discontinuity. For patients discharged home, errors and gaps in care stem from community doctors and care teams who are forced to rely on patient information from the hospital that is incomplete, delayed, or difficult to use.^4,5,6^ These risks are heightened in the context of postacute transitions from hospitals to skilled nursing facilities (SNFs) because they involve a disproportionately high-risk patient population with complex health care needs.^7^ Information discontinuity is considered a significant risk factor for adverse events (eg, medication errors, infections, or even falls) and rehospitalization.^8,9,10,11,12,13,14^ These risks are likely exacerbated by payment policies that increasingly encourage earlier transfer of patients (ie, patients who are less stable) from hospitals to postacute care settings.^15^

Although there is broad consensus that information continuity is important, few national data are available on the current state of information sharing during hospital-SNF care transitions. New incentives that promote stronger hospital-SNF coordination,^16,17^ coupled with a decade of investment in electronic health records (EHRs) and associated information exchange capabilities under the Health Information Technology for Economic and Clinical Health Act,^18^ should leave hospitals well-positioned to share complete, timely, and usable information with SNFs to support care transitions. However, effective use of these tools depends on how hospitals choose to use them; hospitals have discretion over what information they send, when they send it, and how it is structured.^13,19,20^ Hospital to hospital differences in these choices make it challenging for SNFs to locate needed information and to establish standard workflows, which could result in poor transitional care processes and outcomes.^12,21,22^

Therefore, in the present study, we developed and administered a survey to the Directors of Nursing in a national random sample of SNFs that captured, for each of their 2 highest-volume referring hospitals, the completeness, timeliness, and usability of information shared by the hospital to support care transitions. Survey questions were developed with detailed SNF input and included measures of the overall performance on each dimension as well as more specific measures that operationalize each dimension (eg, by asking about receipt of specific data types to measure completeness). Our results offer the first, to our knowledge, national measures of hospital-SNF information sharing, and our detailed measures reveal clear targets for policy and health care system efforts to improve information sharing practices.

## Methods

### Survey Development and Administration

We developed, pilot-tested, revised, and disseminated a survey to the Directors of Nursing in a US national sample of SNFs to capture dimensions of information sharing (completeness, timeliness, and usability) from the SNF perspective. Survey development was informed by qualitative case studies in Age Friendly Health Systems^23^ hospitals and in the SNFs to which they refer patients. We conducted semistructured interviews with 6 hospitals and 12 SNFs (2 per hospital) to understand information sharing practices.^24^ Findings from thematic analysis of interview transcripts helped operationalize our core dimensions of information completeness, timeliness, and usability to translate them into structured questions on our survey instrument. We then pilot tested the survey with the Directors of Nursing in a convenience sample of 6 SNFs in Pennsylvania and Massachusetts, adapting cognitive interviewing techniques to assess question clarity and sequencing.^25^ This study followed the American Association for Public Opinion Research (AAPOR) reporting guideline for survey studies and was approved by the institutional review board at the Harvard T.H. Chan School of Public Health. The approach to informed consent included a statement of consent on the survey cover sheet and language conveying that completion and return of the survey reflected that participants understood the purpose of the study and were willing to participate. All survey respondents were offered an Amazon.com gift card ($100) for completing the survey.

We selected a national random sample of 500 SNFs from the list of SNFs included in the Online Survey Certification and Reporting and the Certification and Survey Provider Enhanced Reporting systems reporting file.^26^ Surveys were mailed to the Director of Nursing of each facility. We chose to survey Directors of Nursing because our case studies revealed that nurses are typically the first to work with hospital information to plan for the care transition and do not have other channels to access hospital information (vs physicians who may be able to log on to the hospital EHR). Therefore, Directors of Nursing typically have insight into information sharing as well as to the broader relational and structural factors included on our survey. We followed up with telephone calls, email, and additional mail between January 2019 and March 2020. The survey could be completed online or by mail, fax, or telephone. The final response rate was 53.0% (following AAPOR Response Rate Option No. 2).^27^ We received responses from 265 SNFs, representing 471 SNF-hospital pairs. Completed surveys were then linked to publicly available data that included organizational characteristics. The SNF characteristics came from the Skilled Nursing Facility Utilization and Payment Public Use File.^28^ The hospital characteristics came from the 2018 American Hospital Association Annual Survey Database and from data publicly reported by the Centers for Medicare & Medicaid Services on hospital participation in bundled payment initiatives that include postacute care services.^26,29^ Finally, we used 2017 Medicare claims data to measure hospital-SNF referral volumes.^30^

### Survey Content

The final survey contained 27 questions organized in 3 sections: hospital relationships, information sharing, and facility and information technology (IT) characteristics. The first 2 sections repeated each question twice—once for each of the 2 acute care hospitals with the highest referral volumes to that SNF. The first section of the survey, “Hospital Relationships,” asked respondents to report on any formal organizational integration (eg, shared ownership, or colocation) with each of the 2 hospitals, as well as informal integration via shared staffing across sites (eg, clinicians or care coordinators), collaboratives or preferred provider networks, and joint participation in performance-related meetings and programs. The second section, “Information Sharing,” asked SNFs to report on the dimensions of information sharing (completeness, timeliness, and usability) and the standard transitional care actions that occurred when they receive patients from each hospital. The final section, “Facility and IT Characteristics,” asked SNFs to report additional information about their facility, including affiliation(s), payer mix, acceptance of patients with additional needs, and health IT infrastructure. The full survey instrument is included in the eAppendix in the Supplement.

### Measures

#### Information Sharing

We first constructed overall performance measures for each dimension of information sharing based on a 5-point Likert scale from 1 (poor) to 5 (excellent) for questions based on responses under the statement “Rate the following dimensions of information sharing [completeness, timeliness, usability] to plan for the transfer of care.” For overall performance measures, we calculated the percentage of hospital-SNF pairs that reported a 5 (ie, excellent) on at least 1 dimension of information sharing, on at least 2 dimensions, and on all 3 dimensions. We then calculated the percentage of pairs at or below mean performance (ie, 3 or below) on 1, on 2, and on all 3 dimensions.

Next, we constructed measures using more detailed questions that corresponded to each dimension. For completeness, we asked: “Do you typically receive the following information about the hospitalization to plan for the transfer of care?” and then listed 23 specific information type categories considered necessary for robust transitional care based on prior research.^26,31,32^ We calculated the percentage of hospital-SNF pairs that reported each of the 23 types of information as not typically received (“missing”). We also calculated the percentage of pairs in which the SNF typically received at least 50% of the 23 information types (at least 12), the percentage that received at least 80% (at least 19), and the percentage that received 100%. Finally, we asked respondents to estimate the number of hours per week spent on back-and-forth communication between the SNF and the hospital to obtain information for patient transfer.

Our detailed timeliness question asked: “When patients are discharged to your facility, how often does discharge information arrive after the patient?” We calculated the percentage of hospital-SNF pairs responding “always/often,” “sometimes,” or “rarely/never.” Using the same scale, our detailed usability question asked: “When patients are discharged to your facility, how often is the discharge documentation: (1) duplicative and/or (2) extraneous?” We also asked whether or not the hospital used a discharge summary that presents information specifically tailored to SNF (or other inpatient postacute care) needs. We calculated the percentage of hospital-SNF pairs in which discharge summaries always or often (1) contained duplicative information, (2) contain extraneous information, and (3) lacked tailoring to SNF needs. Across these 3 measures, we also calculated the percent of SNFs with 0 or 1 usability shortcoming compared with those with 2 or 3 shortcomings.

#### Organizational Factors

We examined relational and structural characteristics that were identified in the literature or emerged during case studies as being associated with the quality of hospital-SNF information sharing. We included 8 measures that described hospital-SNF relationships. Using survey data, we created binary measures of whether the hospital-SNF pair had the following: shared ownership or co-located facilities; informal integration (ie, through shared meetings, programs, or other affiliation via a collaborative or preferred provider relationship); hospital clinicians or care coordinators spanning both sites; SNF staff on site at the hospital; and information sharing via secure text messaging or any form of electronic information access (ie, via shared EHR or access to the hospital’s full inpatient record). Using Medicare claims, we captured the relative importance of the SNF to the hospital using a proxy measure of whether the SNF received at least 25% of the hospitals discharges to SNFs (a >25% cutoff represented approximately the top decile of our distribution).

We also examined structural characteristics of the SNF (4 measures) and hospital (5 measures). For SNFs, those included size (<75 beds, 75-150 beds, or >150 beds), for-profit vs not-for-profit ownership, urbanicity (metro, micro, rural, or small town), and a binary indicator of whether an SNF was in the top quartile of facilities based on the mean Hierarchical Condition Category (HCC) risk score, a patient-level measure of clinical complexity used by Centers for Medicare & Medicaid Services to estimate beneficiaries' spending and perform risk adjustment.^33^ The mean HCC is reported at the facility level in the SNF public use file and can be used to compare patient populations at different facilities. For hospitals, we included size (<100, 100-399, >399 beds), teaching status, and ownership (state government, not-for-profit, for-profit). We also include indicators of whether the hospital participated in an accountable care organization or one of the Centers for Medicare & Medicaid Services bundled payment programs that include postacute care in the bundle to capture hospital incentives to improve postacute transitions and associated outcomes.

### Statistical Analysis

We first compared responding and nonresponding SNFs on structural characteristics to characterize the sample and assess potential nonresponse bias. On the basis of observed differences, we calculated nonresponse weights using a logistic regression model to estimate the likelihood of survey response, with SNF and hospital characteristics as factors. The SNF characteristics in the model included region, ownership, size, the proportion of Medicaid eligible patients, and the proportion of White patients. The hospital characteristics included teaching status, hospital size, and Critical Access Hospital status of the primary referring hospital for the SNF. The final weight for a responding SNF was defined as the inverse of the likelihood of response. We then used those weights to produce nationally representative descriptive statistics, at the hospital-SNF pair level, of our overall performance, and of detailed measures of completeness, timeliness, and usability as described.

Finally, we assessed whether the relational and structural characteristics were associated with better information sharing. We examined 3 information sharing measures from the subset included in our descriptive statistics—whether the SNF typically received from the hospital at least 19 (80%) of the 23 information types (completeness), whether information arrived after the patient rarely/never (timeliness), and whether there was no more than 1 usability shortcoming. We first conducted bivariate models, followed by multivariate logistic regressions, 1 for each information sharing measure, with all characteristics included. All results are presented using odds ratios (ORs). In eTable 1 of the Supplement, we included a table with the response frequency distribution for each relational and structural characteristic across our 3 dimensions of information sharing. We also included a robustness test for our bivariate and multivariate results with clustered standard errors at the SNF level to adjust for multiple observations (ie, 1 SNF responding for 2 hospitals). All analyses were conducted using SAS software, 9.4 (SAS Institute Inc). A 2-sided value of *P* < .05 was considered statistically significant.

## Results

The 265 responding SNFs were similar to the 235 nonresponding SNFs on observed characteristics, with no significant differences in organizational size, profit status, system or chain ownership, or patient demographic characteristics (Table 1).

**Table 1.** Characteristics of Skilled Nursing Facility Respondents

Characteristic	No. (%) of those surveyed		*P* value
	Respondents (n = 265)	Nonrespondents (n = 235)	
Location: census region			
Midwest	34.3	31.9	.35
Northeast	14.3	11.1	
South	34.7	42.1	
West	16.6	14.9	
Location: urbanicity^a^			
Metropolitan area	67.7	69.8	.52
Micropolitan area	12.5	15.1	
Small town	11.8	8.6	
Rural	8.0	6.5	
Ownership			
For profit	69.4	71.5	.06
Government	3.0	6.8	
Nonprofit	27.5	21.7	
Located in hospital	3.4	6.8	.08
Size (bed count)			.44
Large (>125)	29.1	25.1	
Medium (75-125)	43.0	48.5	
Small (<75)	27.9	26.4	
2019 Nursing Home Compare Star Ratings			
Overall rating	3.28	3.17	.37
Quality rating	3.47	3.43	.76
Patient race/ethnicity and insurance status, %			
White	82.7	79.6	.11
Dual eligible	35.0	38.5	.07

^a^ Determined using rural-urban commuting area codes.

### Measures of Information Sharing

**Figure 1.**
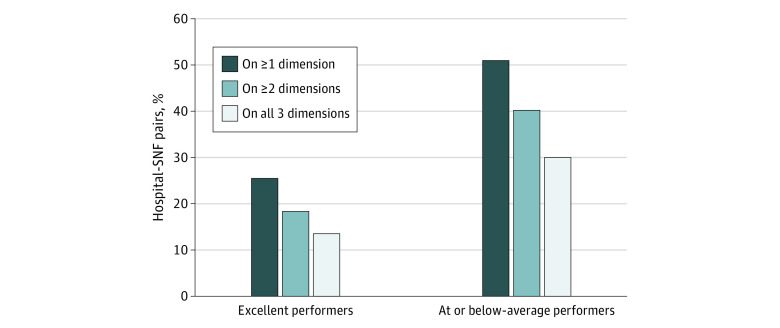
Skilled Nursing Facility (SNF) Ratings: Completeness, Timeliness, and Usability of Information to Support Care Transitions Data are for 471 hospital-SNF pairs. A rating of 5 on a Likert scale of 1 to 5 points indicated excellent performance, whereas a rating of 3 or lower indicated below-average performance.

Respondents (Directors of Nursing) in 64 pairs (13.5%) reported excellent information sharing, with all 3 dimensions rated as 5 of 5. Of 471 hospital-SNF pairs, 141 (30.0%) performed at or below average on all 3 dimensions (Figure 1).

**Figure 2.**
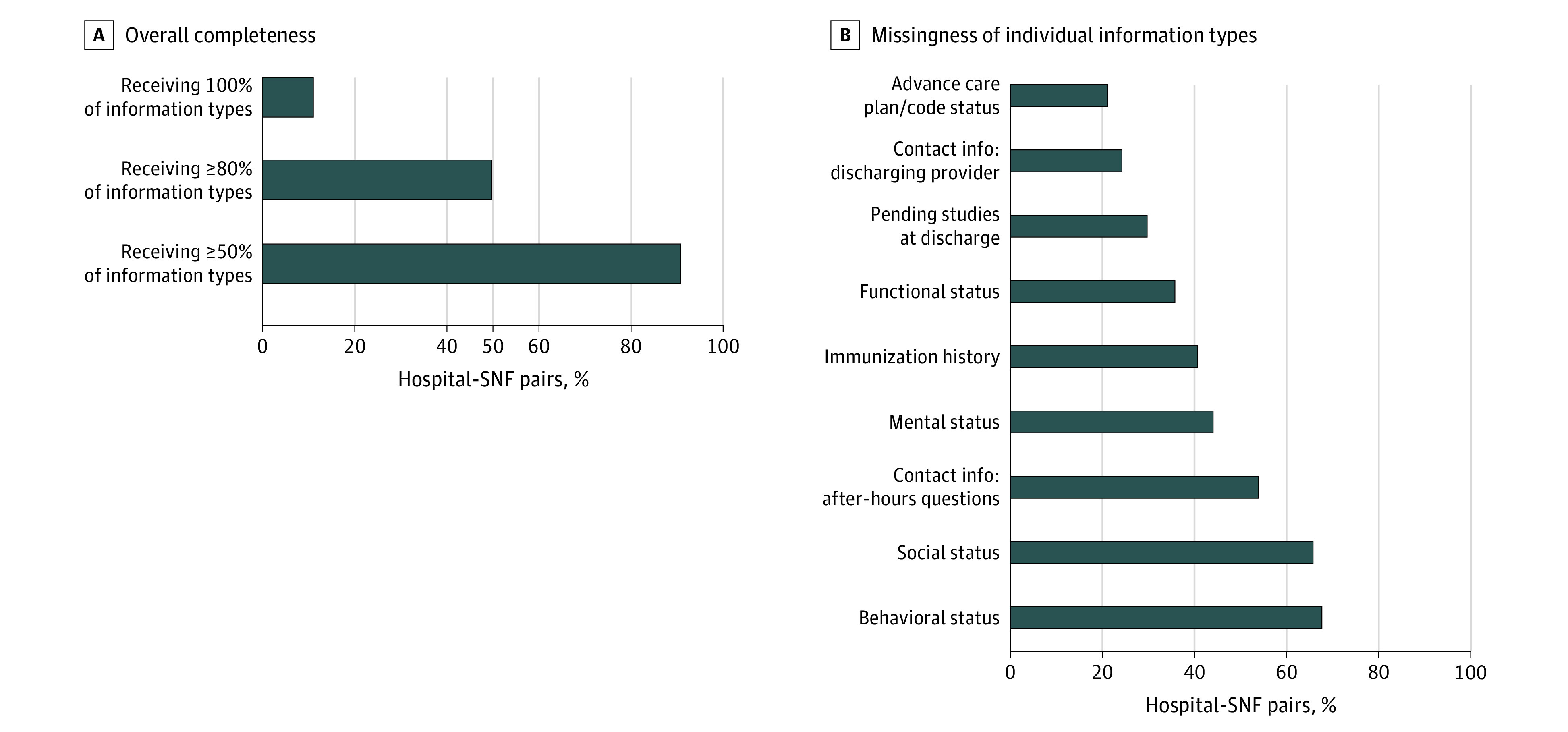
Completeness of Information Received by Skilled Nursing Facilities (SNFs) From Hospitals to Support Care Transitions Right panel includes information elements missing for more than 20% of hospital-SNF pairs. Missingness of all 23 data elements included in the survey is reported in the eAppendix in the Supplement.

The SNF receipt of specific types of information varied widely: 319 hospital-SNF pairs (67.7%) reported behavioral status typically missing compared with only 8 pairs (1.7%) that reported absence of reason for inpatient admission. (Missingness of each type of information is reported in the eFigure in the Supplement.) Half of all hospital-SNF pairs (49.6%) did not meet the bar of at least 19 of the 23 types (80%) of information typically received, and only 52 pairs (11.0%) typically received all 23 information types (Figure 2). Beyond behavioral status, information types that were typically missing included: social status (missing in 309 pairs [65.7%]), hospital contact information for after-hours questions (missing in 254 pairs [53.9%]), mental status (missing in 208 pairs [44.1%]), immunization history (missing in 192 pairs [40.7%]), and functional status/level of independence (missing in 169 pairs [35.8%]). Information types such as code status, contact information for the discharging physician at the hospital, and pending test results were missing in at least 94 pairs (20%). The SNFs reported spending a mean (SD) of 6.5 (8.2) hours per week on back-and-forth communication with the hospital to obtain information.

**Figure 3.**
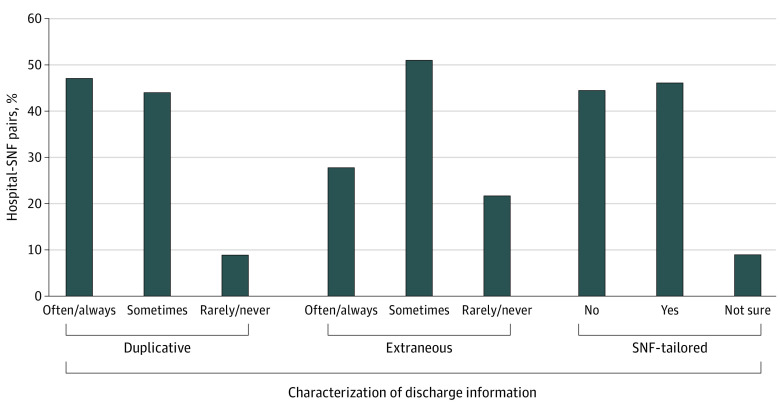
Usability of Information Received by Skilled Nursing Facilities (SNFs) From Hospitals to Support Care Transitions

For timeliness, 77 of hospital-SNF pairs (16.4%) reported that information always or often was received after the patient arrived; an additional 159 pairs (33.8%) reported that this occurred sometimes. For usability, 222 hospital-SNF pairs (47.1%) reported that discharge documentation always or often contained duplicative information and an additional 207 (44.0%) reported that this occurred sometimes (Figure 3). Overall, 131 pairs (27.8%) reported that discharge documentation always or often contained extraneous information and an additional 238 pairs (50.5%) reported “sometimes.” More than half of pairs either lacked (210 [44.5%]) or did not know (44 [9.3%]) whether the hospital offered SNF-tailored discharge information. Although 304 pairs (64.5%) reported none or 1 usability shortcoming across these 3 measures, the remaining 167 pairs (35.5%) had 2 or 3 usability shortcomings.

### Relational and Structural Characteristics Associated With Better Information Sharing

In bivariate analyses, SNF-hospital pairs were more likely to receive more complete information (at least 80% of data types) when they were formally integrated with the hospital (OR, 3.75; 95% CI, 1.62-8.68; *P* = .002), were informally integrated (OR, 2.05; 95% CI, 1.40-2.99; *P* < .001), had hospital clinicians (OR, 1.74; 95% CI, 1.18-2.57; *P* = .005) or care coordinators on site at the SNF (OR, 1.64; 95% CI, 1.10-2.43; *P* = .01), had SNF staff on site at the hospital (OR, 1.69; 95% CI, 1.15-2.47; *P* = .007), and when hospital and SNF staff were able to communicate via text (OR, 1.87; 95% CI, 1.17-3.00; *P* = .01) (Table 2). In terms of structural characteristics, more complete information was positively associated with an SNF being located in a metropolitan area compared with a rural area (OR, 1.96; 95% CI, 1.19-3.22; *P* = .008) and negatively associated with an SNF being in the highest HCC quartile (OR, 0.61; 95% CI, 0.39-0.94; *P* = .03).

**Table 2.** Relational and Structural Characteristics Associated With Better Information Sharing

Relational factor	Odds ratios (95% CI)					
	Completeness: >80% (at least 19 of 23) information elements routinely available		Timeliness: information rarely arriving after patient (compared with sometimes/often/always)		Usability: no more than 1 usability shortcoming (eg, duplicative, extraneous, not tailored to SNF context)	
	Bivariate	Multivariate	Bivariate	Multivariate	Bivariate	Multivariate
Shared ownership or colocation	3.75 (1.62-8.68)^a^	3.17 (1.04-9.65)^b^	1.34 (0.58-3.08)	1.20 (0.44-3.28)	2.53 (0.96-6.64)	2.63 (0.73-9.47)
Informal integration (eg, hospital-SNF collaborative)	2.05 (1.40-2.99)^c^	1.27 (0.77-2.09)	1.01 (0.70-1.48)	1.00 (0.59-1.69)	1.25 (0.85-1.85)	1.05 (0.64-1.71)
Staff spanning hospital and SNF						
Hospital clinicians	1.74 (1.18-2.57)^a^	1.72 (1.07-2.78)^b^	1.55 (1.05-2.30)^b^	1.76 (1.08-2.88)^a^	1.73 (1.16-2.59)^a^	1.64 (1.02-2.63)^b^
Hospital care coordinators	1.64 (1.10-2.43)^b^	1.19 (0.72-1.96)	1.04 (0.70-1.55)	0.91 (0.54-1.53)	1.10 (0.73-1.66)	1.02 (0.61-1.69)
SNF staff on site at hospital	1.69 (1.15-2.47)^a^	1.48 (0.91-2.40)	0.59 (0.40-0.87)^a^	0.54 (0.32-0.90)^b^	0.99 (0.67-1.47)	0.96 (0.59-1.57)
Information sharing technology						
Secure texting	1.87 (1.17-3.00)^a^	1.15 (0.64-2.07)	0.76 (0.48-1.21)	0.83 (0.47-1.47)	1.29 (0.78-2.11)	1.16 (0.62-2.16)
IT integration	1.37 (0.94-1.98)	0.99 (0.61-1.60)	0.74 (0.51-1.08)	0.87 (0.54-1.43)	1.21 (0.82-1.78)	1.26 (0.79-2.01)
Hospital-SNF discharge volume: low vs high^d^	0.89 (0.49-1.64)	1.12 (0.51-2.45)	0.79 (0.43-1.47)	0.45 (0.19-1.06)	0.95 (0.61-1.48)	1.24 (0.56-2.76)
Structural predictors						
HCC top quartile	0.61 (0.39-0.94)^b^	0.59 (0.33-1.05)	0.58 (0.38-0.90)^b^	0.54 (0.30-0.98)^b^	0.95 (0.61-1.48)	0.86 (0.49-1.54)
SNF size						
Large vs small	0.88 (0.53-1.44)	0.94 (0.49-1.82)	1.25 (0.76-2.07)	1.89 (0.95-3.75)	1.00 (0.60-1.67)	1.26 (0.65-2.42)
Medium vs small	1.14 (0.72-1.79)	1.23 (0.67-2.24)	1.02 (0.65-1.61)	1.33 (0.72-2.44)	1.03 (0.64-1.65)	1.29 (0.71-2.34)
Ownership						
For-profit vs nonprofit	0.94 (0.62-1.43)	1.10 (0.62-1.93)	0.46 (0.30-0.70)^c^	0.46 (0.26-0.80)^a^	0.79 (0.51-1.23)	1.06 (0.60-1.86)
Government vs nonprofit	0.67 (0.18-2.55)	0.72 (0.19-2.72)	0.28 (0.07-1.06)	0.16 (0.03-0.93)^b^	0.58 (0.16-2.08)	0.62 (0.16-2.44)
Hospital participation in ACO	1.14 (0.79-1.65)	1.08 (0.66-1.78)	1.51 (1.03-2.19)^b^	1.88 (1.13-3.14)^b^	0.82 (0.56-1.21)	0.71 (0.43-1.18)
Hospital BPCI participation	0.88 (0.53-1.45)	0.77 (0.43-1.39)	0.83 (0.50-1.39)	0.88 (0.46-1.66)	0.62 (0.37-1.02)	0.66 (0.37-1.20)
RUCA						
Metropolitan vs rural/small town	1.96 (1.19-3.22)^a^	2.01 (1.02-3.94)^b^	1.51 (0.92-2.45)	1.66 (0.83-3.33)	1.56 (0.96-2.53)	1.41 (0.72-2.76)
Micropolitan vs rural/small town	1.38 (0.68-2.82)	1.76 (0.78-3.96)	1.14 (0.55-2.33)	0.84 (0.33-2.14)	1.69 (0.81-3.54)	1.53 (0.66-3.55)
Hospital profit status						
For-profit vs government (nonfederal)	1.23 (0.59-2.57)	1.50 (0.59-3.78)	0.94 (0.45-1.97)	0.44 (0.16-1.20)	1.09 (0.52-2.29)	0.99 (0.39-2.49)
Not-for-profit vs government (nonfederal)	1.14 (0.64-2.01)	0.91 (0.48-1.73)	1.23 (0.70-2.17)	0.75 (0.36-1.54)	1.25 (0.71-2.21)	1.31 (0.65-2.64)
Hospital teaching status						
Major teaching vs nonteaching	0.93 (0.53-1.65)	0.63 (0.27-1.48)	1.00 (0.56-1.78)	0.55 (0.23-1.30)	0.81 (0.45-1.45)	0.68 (0.29-1.62)
Minor teaching vs nonteaching	0.83 (0.55-1.26)	0.70 (0.40-1.23)	0.68 (0.45-1.03)	0.42 (0.24-0.72)^a^	0.85 (0.56-1.30)	0.72 (0.42-1.25)
Hospital size						
Small vs large	0.64 (0.37-1.09)	0.48 (0.20-1.11)	0.88 (0.52-1.48)	0.58 (0.24-1.39)	0.89 (0.52-1.51)	0.82 (0.34-1.93)
Medium vs large	0.94 (0.61-1.45)	0.51 (0.27-0.95)^b^	0.97 (0.63-1.50)	0.87 (0.47-1.63)	1.12 (0.71-1.74)	0.81 (0.44-1.47)

Abbreviations: ACO, accountable care organization; BPCI, Bundled Payments for Care Improvement; HCC, Hierarchical Condition Category; IT, information technology; RUCA, rural-urban commuting area; SNF, skilled nursing facility.

^a^ *P* < .01.

^b^ *P* < .05.

^c^ *P* < .001.

^d^ Low volume represents less than 25% of hospital’s SNF discharges; high volume indicates 25% or more of hospital’s SNF discharges.

More timely information (information rarely or never arrives after the patient) was associated with having a hospital clinician on site at the SNF (OR, 1.55; 95% CI, 1.05-2.30; *P* = .03). Hospital-SNF pairs were less likely to have timely information if SNFs had staff on site at the hospital (OR, 0.59; 95% CI, 0.40-0.87; *P* = .007). Structural characteristics associated with more timely information sharing included hospital participation in an accountable care organization (OR, 1.51; 95% CI, 1.03-2.19; *P* = .03), whereas SNFs in the highest HCC quartile (OR, 0.58; 95% CI, 0.38-0.90; *P* = .02) and for-profit SNF ownership (OR, 0.46; 95% CI, 0.30-0.70; *P* = .003) were negatively associated. For usability, the only characteristic associated with no more than 1 usability challenge was having a hospital clinician on site at the SNF (OR, 1.73; 95% CI, 1.16-2.59; *P* = .02).

In multivariate analyses, having a hospital clinician on site at the SNF was the only characteristic that remained statistically significant and was significant for all 3 dimensions: OR, 1.72 (95% CI, 1.07-2.78; *P* = .03) for completeness; OR, 1.76 (95% CI, 1.08-2.88; *P* = .02) for timeliness; and OR, 1.64 (95% CI, 1.02-2.63; *P* = .04) for usability (Table 2). Hospital accountable care organization participation was also significantly associated with improved information continuity in multivariate results but only for timeliness (OR, 1.88; 95% CI, 1.13-3.14; *P* = .02). Our robustness test with clustered standard errors reflected wider confidence intervals but no substantial change in interpretation of findings (eTable 2 in the Supplement). In multivariate results, on-site clinicians remained significantly associated only with timeliness at the *P* < .05 threshold.

## Discussion

We developed and administered a novel national survey to SNF Directors of Nursing to capture detailed measures of information sharing between hospitals and SNFs. Our study findings revealed critical gaps, from the nursing perspective, in the types of information made available during transitions. Information types considered foundational to supporting transitions for every patient (eg, contact information for the discharging hospital provider or pending test results) were missing in at least 20% of hospital-SNF pairs, representing substantial discontinuity that increases risk of patient safety and quality failures.^33,34^ Other information types, such as functional status as well as mental, social, and behavioral health status, were missing at substantially higher rates and are essential to ensuring safe and sufficiently equipped transitions for many patients.^12,20^ For example, for patients with dementia for whom hospital-SNF transitions are common,^35^ SNFs are unable to prepare the necessary staff and patient-centered support services without such information. This insufficient communication adds stress and can destabilize patients who are already vulnerable.^36,37,38^ In addition, SNFs in more than half of pairs in the present study reported delayed information arrival, which can have direct consequences for patients. For example, when pain medication needs are not shared in a timely manner, patients may not be able to receive pain medication for more than 24 hours after discharge. Finally, in three-quarters of pairs, information was duplicative or extraneous or lacked tailoring to the SNF environment. These shortcomings place undue burden on SNFs and compromise SNF efficiency, taking away valuable staff time to review discharge documentation to be adequately prepared for the transition and to ensure that important information is not missed.

Given the shortcomings across all 3 dimensions, our results strongly suggest that hospitals have not sufficiently invested in understanding SNF information needs to support transitional care.^12,13,20,39^ Hospitals have many fronts on which they are being asked to improve care transitions and care coordination, and must make difficult prioritization decisions across them. To address the specific shortcomings we identify, some solutions are likely easier than others. For example, most hospitals use the default discharge summaries included in their EHR and may assume that it is technically complex and expensive to redesign them. However, given that nearly half of our sample reported that the hospital offered an SNF-tailored discharge summary, there are templates that could be shared and deployed more broadly, which would be facilitated by hospital EHR certification criteria that define and include this as a standard template. Improving timeliness is also challenging, given process bottlenecks and competing demands on discharging physicians, who may not even have firsthand knowledge of the patient’s care. Frontline clinicians could be motivated by feedback about their performance and perhaps also rewards for timely completion. However, for timeliness, as well as for determining essential information content for inclusion, a national standard may be necessary to achieve consistency and promote investment in larger process fixes for communication and coordination (such as how to share laboratory results that are returned after discharge or how to identify the clinician responsible for after-hours questions). These investments could have spillover benefits outside of hospital-SNF transitions.

Beyond the need for hospitals to improve individual dimensions of information sharing, our results suggest that having clinicians who span both sites of care may help achieve better information sharing on all 3 dimensions. Although this “SNF-ist” model exists for the explicit purpose of improving care continuity, one of the ways it may do so is by improving information sharing, perhaps by promoting changes in hospitals’ information sharing practices. Shared clinicians are positioned to make the case to hospital leadership of the value of such information to the SNF, and if needed, fill the gap themselves. This includes not only the dimensions of information sharing explored here but also the accuracy and reliability of information that may not be easily assessed by individuals who only see 1 component of the broader care transition. Other forms of formal integration that we expected would be associated with better information sharing—shared ownership and shared IT—were not consistently associated; however, there was only a small number of pairs with these forms, suggesting we were underpowered to detect these associations.

Additional strategies that we hypothesized would be broadly helpful—informal integration (eg, preferred provider relationships or shared participation in meetings or collaboratives) and the use of technology to support communication and information sharing (eg, secure texting or electronic access to the inpatient EHR)—were only weakly associated with better information sharing, and only for completeness. Similarly, hospital accountable care organization participation was associated only for 1 dimension—timeliness. These findings are consistent with other work suggesting that current reforms promoting tighter hospital-SNF relationships are not systematically resulting in improved coordination nor improvements in patient outcomes.^34,40^ Taken together, our results point to the need for stronger policy actions that either directly require improved information sharing by hospitals, or further increase hospital prioritization of postacute transitions via delivery or payment reform levers.

### Limitations

Our study has several limitations. First, our survey asked SNFs only about information sharing from the 2 hospitals from which they receive the most patients. These are likely to be prioritized relationships with the strongest processes in place. Thus, although our results identify major shortcomings in information sharing, they likely overstate the quality of information sharing between hospitals and SNFs and do not examine all dimensions of information quality (eg, accuracy and reliability). Our results also do not assess the relationships between information sharing and outcomes. Second, our study uses self-reported survey data, which we were not able to independently validate. Although we tried to address this with a range of questions, the majority of which asked about objective constructs, all responses were subject to the perception of the individual completing the survey. Relatedly, we captured perspectives specifically among SNF Directors of Nursing who—although they are assumed to have a broadly informed view of information sharing practices—may assess information sharing differently than frontline nursing staff, physicians, or other types of clinicians who support transitional care in SNFs. Third, our approaches to adjust for nonresponse bias may not have adequately addressed differences between respondents and nonrespondents although the lack of differences on observed characteristics prior to adjusting is encouraging. In addition, our sample represented only about 5% of SNFs in the nation and may have been underpowered to detect associations across the many relational and structural characteristics that we examined. Fourth, examining many structural and relational characteristics for 3 different dimensions of information sharing could increase the likelihood of false-positives. This led us to focus results reporting and interpretation on characteristics that were significant across multiple models (ie, shared clinicians).

## Conclusions

In a US nationally representative survey of SNFs, shortcomings in the completeness, timeliness, and usability of information provided by the 2 largest referral volume hospitals to support postacute transitions were found. Clinicians spanning both sites of care was the only significant factor associated with better information sharing across all 3 dimensions, suggesting a near-term mechanism that could improve information sharing. However, broader hospital-led efforts are likely needed, ideally supported by ongoing efforts to improve IT infrastructure and align incentives behind high-quality care transitions.

## Notes

Supplement. **eTable 1.** Frequency Table Reporting Distribution of Respondents, by Covariate and Across Outcomes **eFigure.** Reported Missingness of Each Information Type to Support Hospital-SNF Care Transitions (All Types Included on Survey) **eTable 2.** Relational and Structural Characteristics Associated With Better Information Sharing (Odds Ratios & 95% CIs), Clustered (SNF-Level) Standard Errors **eAppendix.** Survey Instrument
