# Supplementary material for: Information Sharing Practices Between US Hospitals and Skilled Nursing Facilities to Support Care Transitions
Source: JAMA Netw Open. 2021 Jan 14;4(1):e2033980. doi: 10.1001/jamanetworkopen.2020.33980 (PMC7809587; doi:10.1001/jamanetworkopen.2020.33980)
Supplement: Supplement. — eTable 1. Frequency Table Reporting Distribution of Respondents, by Covariate and Across Outcomes eFigure. Reported Missingness of Each Information Type to Support Hospital-SNF Care Transitions (All Types Included on Survey) eTable 2. Relational and Structural Characteristics Associated With Better Information Sharing (Odds Ratios & 95% CIs), Clustered (SNF-Level) Standard Errors eAppendix. Survey Instrument [file jamanetwopen-e2033980-s001.pdf]

Supplementary Online Content

Adler-Milstein J, Raphael K, O'Malley TA, Cross DA. Information sharing practices between US hospitals and skilled nursing facilities to support care transitions. *JAMA Netw Open*. 2021;4(1):e2033980. doi:10.1001/jamanetworkopen.2020.33980

eTable 1. Frequency Table Reporting Distribution of Respondents, by Covariate and Across Outcomes

eFigure. Reported Missingness of Each Information Type to Support Hospital-SNF Care Transitions (All Types Included on Survey)

eTable 2. Relational and Structural Characteristics Associated with Better Information Sharing (Odds Ratios & 95% CIs), Clustered (SNF-Level) Standard Errors

eAppendix. Survey Instrument

This supplementary material has been provided by the authors to give readers additional information about their work.

© 2021 Adler-Milstein J. *JAMA Network Open*.

eTable 1. Frequency Table Reporting Distribution of Respondents, by Covariate and Across Outcomes

	Completeness: Regularly receives 80% of identified data elements (max n = 464)				Timeliness: Information rarely/never arrives after the patient (max n = 464)				Usability: SNF reports no more than 1 usability shortcoming (max n = 470)			
	Yes (max n = 228)		No (max n = 240)		Yes (max n = 228)		No (max n = 236)		Yes (max n = 300)		No (max n = 170)	
**In Hospital/Shared Ownership (from survey)**												
Yes	18	(8%)	9	(4%)	15	(7%)	12	(5%)	21	(7%)	6	(4%)
No	195	(86%)	221	(92%)	203	(89%)	208	(88%)	260	(87%)	157	(92%)
Missing	15	(7%)	10	(4%)	10	(4%)	16	(7%)	19	(6%)	7	(4%)
**Informal Integration (from survey)**												
Yes	141	(62%)	116	(48%)	125	(55%)	127	(54%)	168	(56%)	88	(52%)
No	82	(36%)	124	(52%)	102	(45%)	105	(44%)	128	(43%)	81	(48%)
Missing	5	(2%)	0	(0%)	1	(0%)	4	(2%)	4	(1%)	1	(1%)
**Shared Clinician (from survey)**												
Yes	106	(46%)	87	(36%)	109	(48%)	82	(35%)	134	(45%)	59	(35%)
No	108	(47%)	141	(59%)	111	(49%)	136	(58%)	144	(48%)	107	(63%)
Missing	14	(6%)	12	(5%)	8	(4%)	18	(8%)	22	(7%)	4	(2%)
**Shared Care Coordinators (from survey)**												
Yes	93	(41%)	74	(31%)	84	(37%)	83	(35%)	109	(36%)	58	(34%)
No	126	(55%)	154	(64%)	132	(58%)	144	(61%)	176	(59%)	106	(62%)
Missing	9	(4%)	12	(5%)	12	(5%)	9	(4%)	15	(5%)	6	(4%)
**Info Share A - SNF Staff On Site (from survey)**												
Yes	143	(63%)	124	(52%)	115	(50%)	146	(62%)	167	(56%)	98	(58%)
No	82	(36%)	113	(47%)	111	(49%)	86	(36%)	127	(42%)	71	(42%)
Missing	3	(1%)	3	(1%)	2	(1%)	4	(2%)	6	(2%)	1	(1%)
**Info Share C - Texting (from survey)**												

© 2021 Adler-Milstein J. *JAMA Network Open*.

Yes	55	(24%)	37	(15%)	40	(18%)	53	(22%)	64	(21%)	29	(17%)
No	169	(74%)	201	(84%)	187	(82%)	179	(76%)	233	(78%)	138	(81%)
Missing	4	(2%)	2	(1%)	1	(0%)	4	(2%)	3	(1%)	3	(2%)
**Info Share F/H - IT Integration (from survey)**												
Yes	125	(55%)	119	(50%)	110	(48%)	132	(56%)	160	(53%)	84	(49%)
No	102	(45%)	120	(50%)	118	(52%)	103	(44%)	139	(46%)	85	(50%)
Missing	1	(0%)	1	(0%)	0	(0%)	1	(0%)	1	(0%)	1	(1%)
**Transfer Relationship (from 2016 claims)**												
Low/Medium	201	(88%)	203	(85%)	192	(84%)	207	(88%)	259	(86%)	145	(85%)
High	25	(11%)	25	(10%)	27	(12%)	23	(10%)	30	(10%)	20	(12%)
Missing	2	(1%)	12	(5%)	9	(4%)	6	(3%)	11	(4%)	5	(3%)
**HCC Top Quartile**												
Top Quartile	46	(20%)	70	(29%)	47	(21%)	70	(30%)	72	(24%)	45	(26%)
Not Top Quartile	180	(79%)	170	(71%)	178	(78%)	165	(70%)	225	(75%)	124	(73%)
Missing	2	(1%)	0	(0%)	3	(1%)	1	(0%)	3	(1%)	1	(1%)
**SNF Size**												
Small	61	(27%)	66	(28%)	57	(25%)	71	(30%)	82	(27%)	47	(28%)
Medium	103	(45%)	97	(40%)	97	(43%)	103	(44%)	130	(43%)	72	(42%)
Large	64	(28%)	77	(32%)	74	(32%)	62	(26%)	88	(29%)	51	(30%)
**SNF Ownership**												
For Profit	162	(71%)	166	(69%)	145	(64%)	179	(76%)	207	(69%)	123	(72%)
Non-profit	62	(27%)	67	(28%)	79	(35%)	50	(21%)	88	(29%)	41	(24%)
Government	4	(2%)	7	(3%)	4	(2%)	7	(3%)	5	(2%)	6	(4%)
**Hospital BPCI participation**												
Participating	35	(15%)	41	(17%)	32	(14%)	41	(17%)	42	(14%)	34	(20%)
Not Participating	193	(85%)	199	(83%)	196	(86%)	195	(83%)	258	(86%)	136	(80%)
**RUCA**												

© 2021 Adler-Milstein J. *JAMA Network Open*.

Metropolitan	173	(76%)	152	(63%)	168	(74%)	156	(66%)	217	(72%)	112	(66%)
Micropolitan	23	(10%)	28	(12%)	22	(10%)	27	(11%)	33	(11%)	16	(9%)
Rural/Small Town	32	(14%)	58	(24%)	36	(16%)	53	(22%)	49	(16%)	41	(24%)
Missing	0	(0%)	2	(1%)	2	(1%)	0	(0%)	1	(0%)	1	(1%)
**Hospital Profit Status**												
For Profit	30	(13%)	30	(13%)	26	(11%)	33	(14%)	35	(12%)	24	(14%)
Not for profit	171	(75%)	177	(74%)	174	(76%)	171	(72%)	230	(77%)	120	(71%)
Gov (non-federal)	27	(12%)	33	(14%)	28	(12%)	32	(14%)	35	(12%)	26	(15%)
**Hospital Teaching Status**												
Major Teaching	30	(13%)	31	(13%)	31	(14%)	29	(12%)	38	(13%)	24	(14%)
Minor Teaching	74	(32%)	84	(35%)	69	(30%)	90	(38%)	100	(33%)	60	(35%)
Non-teaching	124	(54%)	125	(52%)	128	(56%)	117	(50%)	162	(54%)	86	(51%)
**Hospital Size**												
Small	40	(18%)	61	(25%)	46	(20%)	54	(23%)	60	(20%)	41	(24%)
Medium	114	(50%)	112	(47%)	110	(48%)	112	(47%)	148	(49%)	77	(45%)
Large	74	(32%)	67	(28%)	72	(32%)	70	(30%)	92	(31%)	52	(31%)

© 2021 Adler-Milstein J. *JAMA Network Open*.

eFigure. Reported Missingness of Each Information Type to Support Hospital-SNF Care Transitions (All Types Included on Survey)

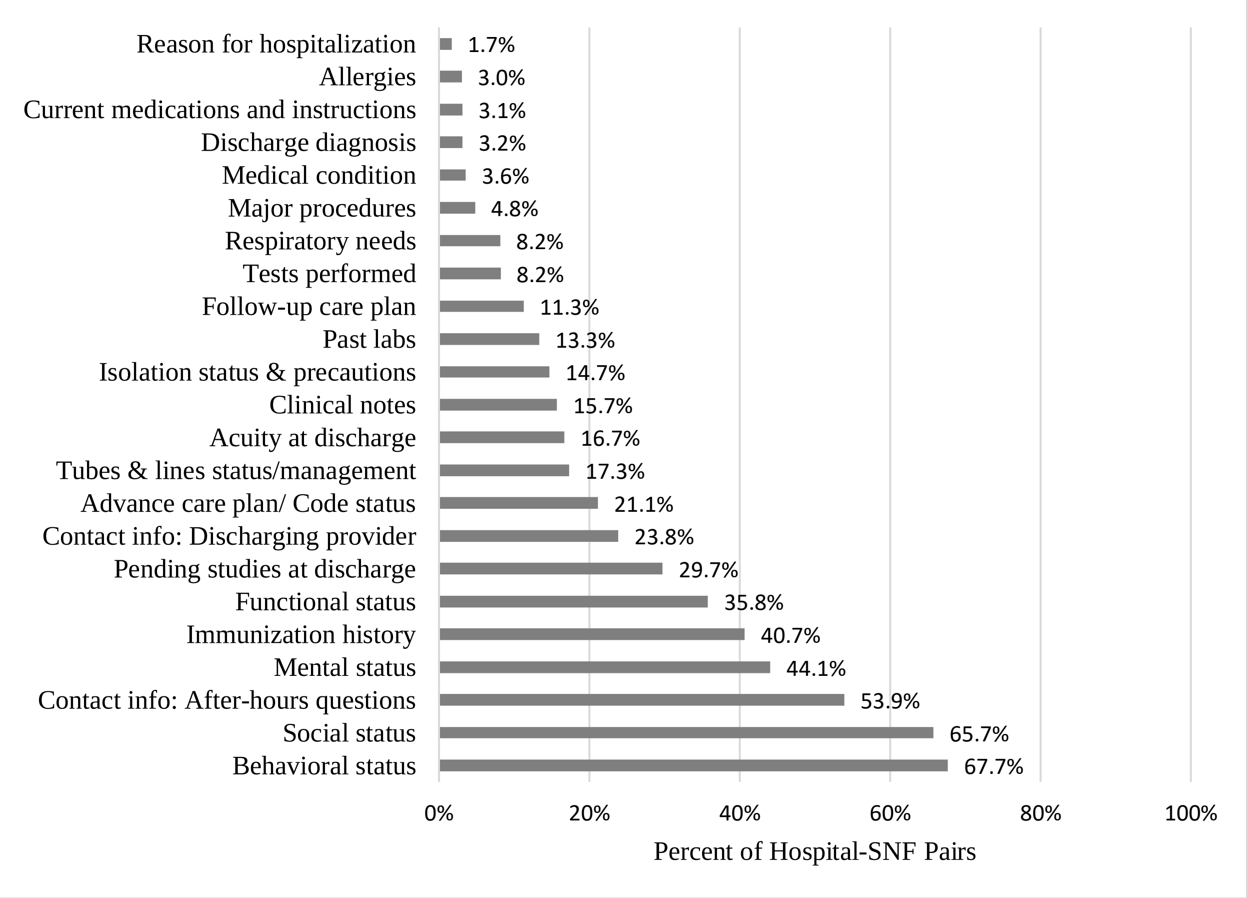

© 2021 Adler-Milstein J. *JAMA Network Open*.

eTable 2. Relational and Structural Characteristics Associated with Better Information Sharing (Odds Ratios & 95% CIs), Clustered (SNF-Level) Standard Errors

	**Completeness:** >80% (at least 19 out of 23) information elements routinely available		**Timeliness:** Information rarely arriving after the patient (compared to sometimes/ often/always)		**Usability:** No more than one usability shortcoming (e.g., duplicative, extraneous, not tailored to SNF context)	
Relational Predictors	Bivariate	Multivariate	Bivariate	Multivariate	Bivariate	Multivariate
Shared Ownership and/or Co-location	3.75 (1.47, 9.55)**	3.17 (0.87, 11.53)	1.34 (0.53, 3.38)	1.20 (0.40, 3.62)	2.53 (0.94, 6.83)	2.63 (0.69, 9.98)
Informal Integration (e.g. hospital-SNF collaborative)	2.05 (1.31, 3.20)**	1.27 (0.70, 2.30)	1.01 (0.67, 1.54)	1.00 (0.54, 1.83)	1.25 (0.81, 1.94)	1.05 (0.60, 1.85)
Staff that Span Hospital and SNF:						
Hospital Clinicians	1.74 (1.11, 2.73)*	1.72 (0.98, 3.02)	1.55 (0.99, 2.44)	1.76 (1.03, 3.02)*	1.73 (1.08, 2.77)*	1.64 (0.94, 2.84)
Hospital Care Coordinators	1.64 (0.99, 2.70)	1.19 (0.65, 2.17)	1.04 (0.65, 1.69)	0.91 (0.49, 1.70)	1.10 (0.67, 1.82)	1.02 (0.54, 1.89)
SNF Staff On-Site at Hospital	1.69 (1.05, 2.70)*	1.48 (0.83, 2.64)	0.59 (0.37, 0.94)*	0.54 (0.29, 0.98)*	0.99 (0.62, 1.60)	0.96 (0.54, 1.73)
Info Sharing Technology:						
Secure Texting	1.87 (1.07, 3.28)*	1.15 (0.58, 2.27)	0.76 (0.42, 1.36)	0.83 (0.42, 1.64)	1.29 (0.71, 2.33)	1.16 (0.56, 2.41)
IT Integration	1.37 (0.87, 2.14)	0.99 (0.56, 1.76)	0.74 (0.47, 1.15)	0.87 (0.50, 1.52)	1.21 (0.76, 1.93)	1.26 (0.72, 2.20)
Hospital-SNF Discharge Volume: Low vs. High:[1]	0.89 (0.47, 1.69)	1.12 (0.48, 2.63)	0.79 (0.42, 1.49)	0.45 (0.18, 1.12)	1.04 (0.56, 1.95)	1.24 (0.55, 2.79)
Structural Predictors						
HCC Top Quartile	0.61 (0.34, 1.07)	0.59 (0.28, 1.25)	0.58 (0.33, 1.03)	0.54 (0.26, 1.14)	0.95 (0.53, 1.70)	0.86 (0.42, 1.80)
SNF Size - Large vs Small	0.88 (0.47, 1.65)	0.94 (0.42, 2.13)	1.25 (0.64, 2.45)	1.89 (0.79, 4.49)	1.00 (0.51, 1.96)	1.26 (0.54, 2.93)
SNF Size - Medium vs Small	1.14 (0.62, 2.09)	1.23 (0.57, 2.65)	1.02 (0.56, 1.86)	1.33 (0.62, 2.83)	1.03 (0.55, 1.92)	1.29 (0.60, 2.76)
Ownership: For-Profit vs Non-Profit	0.94 (0.54, 1.63)	1.10 (0.53, 2.28)	0.46 (0.26, 0.81)**	0.46 (0.22, 0.94)*	0.79 (0.45, 1.39)	1.06 (0.52, 2.16)
Ownership: Government vs Non-Profit	0.67 (0.19, 2.33)	0.72 (0.19, 2.71)	0.28 (0.07, 1.14)	0.16 (0.03, 1.06)	0.58 (0.10, 3.20)	0.62 (0.11, 3.53)

© 2021 Adler-Milstein J. *JAMA Network Open*.

Hospital Participation in ACO	1.14 (0.79, 1.65)	1.08 (0.65, 1.81)	1.51 (1.03, 2.21)*	1.88 (1.11, 3.19)*	0.82 (0.56, 1.22)	0.71 (0.44, 1.16)
Hospital BPCI participation	0.88 (0.52, 1.48)	0.77 (0.42, 1.39)	0.83 (0.48, 1.43)	0.88 (0.44, 1.74)	0.62 (0.37, 1.04)	0.66 (0.36, 1.23)
RUCA - Metropolitan vs Rural/Small Town	1.96 (1.03, 3.74)*	2.01 (0.86, 4.66)	1.51 (0.81, 2.81)	1.66 (0.72, 3.86)	1.56 (0.82, 2.95)	1.41 (0.60, 3.29)
RUCA - Micropolitan vs Rural/Small Town	1.38 (0.53, 3.58)	1.76 (0.63, 4.97)	1.14 (0.44, 2.96)	0.84 (0.26, 2.73)	1.69 (0.67, 4.24)	1.53 (0.54, 4.29)
Hospital Profit Status - For-profit vs Government (non-federal)	1.23 (0.57, 2.65)	1.50 (0.57, 3.91)	0.94 (0.43, 2.04)	0.44 (0.15, 1.25)	1.09 (0.51, 2.35)	0.99 (0.39, 2.51)
Hospital Profit Status - Not-for-Profit vs Government (non-federal)	1.14 (0.61, 2.11)	0.91 (0.47, 1.77)	1.23 (0.68, 2.22)	0.75 (0.35, 1.58)	1.25 (0.70, 2.23)	1.31 (0.67, 2.59)
Hospital Teaching Status - Major Teaching vs Non-teaching	0.93 (0.52, 1.66)	0.63 (0.26, 1.53)	1.00 (0.56, 1.79)	0.55 (0.22, 1.35)	0.81 (0.45, 1.44)	0.68 (0.29, 1.62)
Hospital Teaching Status - Minor Teaching vs Non-teaching	0.83 (0.53, 1.31)	0.70 (0.38, 1.30)	0.68 (0.44, 1.06)	0.42 (0.23, 0.76)**	0.85 (0.56, 1.30)	0.72 (0.42, 1.25)
Hospital Size - Small vs Large (reference)	0.64 (0.36, 1.13)	0.48 (0.20, 1.16)	0.88 (0.50, 1.54)	0.58 (0.23, 1.47)	0.89 (0.50, 1.57)	0.82 (0.34, 1.94)
Hospital Size - Medium vs Large (reference)	0.94 (0.62, 1.44)	0.51 (0.27, 0.96)*	0.97 (0.63, 1.50)	0.87 (0.45, 1.69)	1.12 (0.73, 1.71)	0.81 (0.43, 1.52)

Notes: * p-value <0.05; ** p <0.01; *** p<0.001; [1] Low volume=<25% of hospital's SNF discharges; High volume= >25% of hospital's SNF discharges.

© 2021 Adler-Milstein J. *JAMA Network Open*.

eAppendix. Survey Instrument

Hospital Relationships

We identified the acute-care hospitals below as the two facilities from which you receive the largest volume of patients. If this is inaccurate, please cross out and write-in the names of up to two acute-care hospitals from which you receive the most patients.

	Hospital Name 1	Hospital Name 2
**Monthly shared patient volume (average)**		
**Organizational relationship:**		
Shared ownership	Yes ☐ No ☐	Yes ☐ No ☐
Formal affiliation or preferred provider	Yes ☐ No ☐	Yes ☐ No ☐
Co-located (i.e., shared facility)	Yes ☐ No ☐	Yes ☐ No ☐
Participate in hospital's SNF collaborative	Yes ☐ No ☐	Yes ☐ No ☐
Participate in shared pay-for-performance or quality/value program	Yes ☐ No ☐	Yes ☐ No ☐
**Do you have any supervising clinicians who follow patients between the hospital and your facility** (physicians, nurse practitioners, or physician assistants)?	Yes ☐ No ☐	Yes ☐ No ☐
If yes, proportion of your skilled patients covered by shared clinicians	%	%
**Do you have any care coordinators or case managers who follow patients between the hospital and your facility?**	Yes ☐ No ☐	Yes ☐ No ☐
**Do you have meetings with clinical and/or administrative leadership from the hospital and your facility?** (for example, as part of a SNF collaborative)	Yes ☐ No ☐	Yes ☐ No ☐
If yes, number of meetings per year	per year	per year
**Do you engage in shared quality improvement activities?** (for example, shared dashboards or adverse event reviews)	Yes ☐ No ☐ Don't know ☐	Yes ☐ No ☐ Don't know ☐
**Do you have any shared care pathways or protocols?** (for example, shared program to identify/treat high-risk patients)	Yes ☐ No ☐ Don't know ☐	Yes ☐ No ☐ Don't know ☐
**Do you have any shared processes for improving medication safety?** (for example, hospital pharmacist talks to SNF staff)	Yes ☐ No ☐ Don't know ☐	Yes ☐ No ☐ Don't know ☐
**Do you have any shared processes for avoiding hospital or ED visits for residents while they are at your facility?** (for example, hospital does telemonitoring or telehealth)	Yes ☐ No ☐ Don't know ☐	Yes ☐ No ☐ Don't know ☐
**Do you use any transitional care tools?** (for example, INTERACT tools)	Yes ☐ No ☐ Don't know ☐	Yes ☐ No ☐ Don't know ☐

Information Sharing

Please think about the patients you have received from Hospital Name 1 and Hospital Name 2 over the past month.

For these patients, you received information from these hospitals to:
(1) make a REFERRAL DECISION
(2) plan for the TRANSFER OF CARE

Please answer the following questions about INFORMATION RECEIVED from the hospital (as part of referral decision, between referral decision and transfer, or during the transfer itself) to plan for the TRANSFER OF CARE to the NURSING TEAM at your facility who will care for the patient.

1. Please rate the following dimensions of information sharing to plan for the transfer of care:

	Hospital Name 1					Hospital Name 2				
	Poor (1)	(2)	(3)	(4)	Excellent (5)	Poor (1)	(2)	(3)	(4)	Excellent (5)
a. **Completeness**: *All needed information is routinely provided*	☐	☐	☐	☐	☐	☐	☐	☐	☐	☐
b. **Timeliness**: *Information is provided in a timely manner to support decision making*	☐	☐	☐	☐	☐	☐	☐	☐	☐	☐
c. **Usability**: *Information is easy to find, understandable, etc.*	☐	☐	☐	☐	☐	☐	☐	☐	☐	☐

2. Please indicate how often you use the following approaches to receive information to plan for the transfer of care:

	Hospital Name 1			Hospital Name 2		
	Always/ Often	Sometimes	Rarely/ Never	Always/ Often	Sometimes	Rarely/ Never
a. SNF staff onsite at hospital	☐	☐	☐	☐	☐	☐
b. Phone conversation with hospital staff	☐	☐	☐	☐	☐	☐
c. Secure texting with hospital staff	☐	☐	☐	☐	☐	☐
d. Fax/eFax to inbox or portal	☐	☐	☐	☐	☐	☐
e. Shared online referral platform	☐	☐	☐	☐	☐	☐
f. Shared electronic medical record	☐	☐	☐	☐	☐	☐
g. Online portal to view discharge document in hospital EMR	☐	☐	☐	☐	☐	☐
h. Online portal to view full inpatient record in hospital EMR	☐	☐	☐	☐	☐	☐

© 2021 Adler-Milstein J. *JAMA Network Open*.

i. Carried by patient, caregiver, or EMS	☐	☐	☐	☐	☐	☐
j. Other: ______________________________ _	☐	☐	☐	☐	☐	☐

3. When patients are discharged to your facility, which of these transitional care actions are *standard process*?

	**Hospital Name 1**			**Hospital Name 2**		
	**Always/ Often**	**Sometimes**	**Rarely/ Never**	**Always/ Often**	**Sometimes**	**Rarely/ Never**
a. Phone call from discharge planner to SNF clinical staff member or administrator	☐	☐	☐	☐	☐	☐
b. Phone call from floor nurse to SNF clinical staff member	☐	☐	☐	☐	☐	☐
c. Phone call from hospital physician to SNF physician	☐	☐	☐	☐	☐	☐
d. Secure text message or email-type message from hospital to SNF	☐	☐	☐	☐	☐	☐
e. Other:___________________________________	☐	☐	☐	☐	☐	☐

4. Do you typically receive the following information about the hospitalization to plan for the transfer of care?

	**Hospital Name 1**	**Hospital Name 2**
a. Reason for inpatient admission	Yes ☐ No ☐	Yes ☐ No ☐
b. Principal diagnosis at discharge	Yes ☐ No ☐	Yes ☐ No ☐
c. Medical condition	Yes ☐ No ☐	Yes ☐ No ☐
d. Acuity at discharge	Yes ☐ No ☐	Yes ☐ No ☐
e. Current medications and instructions (including IV medications)	Yes ☐ No ☐	Yes ☐ No ☐
f. Allergies	Yes ☐ No ☐	Yes ☐ No ☐
g. Major procedures	Yes ☐ No ☐	Yes ☐ No ☐
h. Tests performed	Yes ☐ No ☐	Yes ☐ No ☐
i. Clinical notes	Yes ☐ No ☐	Yes ☐ No ☐
j. Past labs	Yes ☐ No ☐	Yes ☐ No ☐
k. Pending studies at discharge	Yes ☐ No ☐	Yes ☐ No ☐
l. Contact information for discharging provider	Yes ☐ No ☐	Yes ☐ No ☐
m. Contact information for after-hours questions	Yes ☐ No ☐	Yes ☐ No ☐
n. Follow-up care plan (including appointments)	Yes ☐ No ☐	Yes ☐ No ☐

© 2021 Adler-Milstein J. *JAMA Network Open*.

o. Advanced care plan, health care proxy, and code status/MOLST	Yes ☐ No ☐	Yes ☐ No ☐
p. Immunization history	Yes ☐ No ☐	Yes ☐ No ☐
q. Tubes & lines status/management (e.g., chest x-ray for central lines)	Yes ☐ No ☐	Yes ☐ No ☐
r. Respiratory needs	Yes ☐ No ☐	Yes ☐ No ☐
s. Isolation status & precautions	Yes ☐ No ☐	Yes ☐ No ☐
t. Patient inclusion in ACO, bundled payment, and/or condition-specific protocols	Yes ☐ No ☐	Yes ☐ No ☐

5. How often do you receive **complete information, without having to request it or follow-up for missing information,** to plan for the transfer of care?

	Hospital Name 1			Hospital Name 2		
	**Always/ Often**	**Sometimes**	**Rarely/ Never**	**Always/ Often**	**Sometimes**	**Rarely/ Never**
a. Functional status and level of independence (including ambulatory and diet status)	☐	☐	☐	☐	☐	☐
b. Mental status	☐	☐	☐	☐	☐	☐
c. Behavioral status	☐	☐	☐	☐	☐	☐
d. Social status	☐	☐	☐	☐	☐	☐

6. Are there other types of information not listed above that would be useful to plan for the transfer of care but are not routinely received? If so, please list:

Hospital Name 1	Hospital Name 2

7. How much back-and-forth communication is required to obtain needed information to plan for the transfer of care?

Hospital Name 1					Hospital Name 2				
**Minimal**		**Moderate**		**Substantial**	**Minimal**		**Moderate**		**Substantial**
**(1)**	**(2)**	**(3)**	**(4)**	**(5)**	**(1)**	**(2)**	**(3)**	**(4)**	**(5)**
☐	☐	☐	☐	☐	☐	☐	☐	☐	☐

8. When patients are discharged to your facility, how often is the discharge documentation:

	Hospital Name 1	Hospital Name 2

© 2021 Adler-Milstein J. *JAMA Network Open*.

	Always/ Often	Sometimes	Rarely/ Never	Always/ Often	Sometimes	Rarely/ Never
a. **Sections clearly labeled** (e.g., H&P, med list, labs, consults)	☐	☐	☐	☐	☐	☐
b. **Sections presented in the same order** (i.e., standard format)	☐	☐	☐	☐	☐	☐
c. **Most useful information for nursing team** (e.g., last dose of each medication) **presented first or bolded/highlighted**	☐	☐	☐	☐	☐	☐
d. **Problems and solutions co-located** (e.g., if patient had UTI, medication regimen and current status listed on same line)	☐	☐	☐	☐	☐	☐
e. **Formatted for online and print viewing**	☐	☐	☐	☐	☐	☐
f. **Duplicative** (i.e., same information located in multiple places)	☐	☐	☐	☐	☐	☐
g. **Extraneous** (i.e., includes information that isn't valuable)	☐	☐	☐	☐	☐	☐
h. Other: ______________________	☐	☐	☐	☐	☐	☐

9. Does the hospital use a discharge summary that presents information specifically tailored to SNF (or other inpatient PAC) needs?

Hospital Name 1		Hospital Name 2	
**Yes**	**No**	**Yes**	**No**
☐	☐	☐	☐

10. When patients are discharged to your facility, how often does discharge information arrive AFTER the patient:

Hospital Name 1			Hospital Name 2		
**Always/Often**	**Sometimes**	**Rarely/Never**	**Always/Often**	**Sometimes**	**Rarely/Never**
☐	☐	☐	☐	☐	☐

11. Are patients' medication and equipment needs communicated in a timely fashion such that they can be ordered in advance when appropriate/allowed?

Hospital Name 1			Hospital Name 2		
**Always/Often**	**Sometimes**	**Rarely/Never**	**Always/Often**	**Sometimes**	**Rarely/Never**
☐	☐	☐	☐	☐	☐

© 2021 Adler-Milstein J. *JAMA Network Open*.

12. Please estimate how many **hours per week** are typically spent addressing patient information sharing needs (e.g., finding missing information, obtaining late documents, going through poorly organized or overly long documents) for discharged patients?

Hospital Name 1	Hospital Name 2

© 2021 Adler-Milstein J. *JAMA Network Open*.

Facility and IT Characteristics

1. Is your facility part of a larger organization, such as a corporate chain)?

	Yes	No
	☐	☐

If yes, name of parent organizati[illegible] [ ]

2. Is your facility associated or affiliated with an academic medical center?

Yes, formally ☐ **Yes, informally** ☐ **No** ☐

3. What is your payer mix (% of patients in your facility in a typical month)?

   a. **Medicaid** _____%
   b. **Medicare** (FFS or Medicare Advantage) _____%
   c. **Private** _____%
   d. **Other** _____%

4. How routinely do you accept patients with:

	Always/Often	Sometimes	Rarely/Never
a. **Ventilators**	☐	☐	☐
b. **Requiring isolation**	☐	☐	☐
c. **Obesity** (e.g., bariatric equipment)	☐	☐	☐
d. **Serious mental illness**	☐	☐	☐
e. **Medication assisted treatment** (e.g., buprenorphine)	☐	☐	☐
f. **Substance use disorders**	☐	☐	☐

5. To what extent does your facility **currently use a computerized system** to perform the following tasks:

	(1) Fully electronic	(2) Part electronic-Part paper	(3) All Paper	(4) Don't know
a. Documenting clinical notes from facility staff	☐	☐	☐	☐
b. Viewing lab results	☐	☐	☐	☐
c. Viewing imaging reports	☐	☐	☐	☐
d. Entering medication orders	☐	☐	☐	☐

© 2021 Adler-Milstein J. *JAMA Network Open*.

	(1) Fully electronic	(2) Part electronic-Part paper	(3) All Paper	(4) Don't know
e. Bar-coded medication administration	☐	☐	☐	☐
f. Clinical decision support for medication (e.g., alerts for wrong dose)	☐	☐	☐	☐

6. What is your primary EMR vendor?

PointClickCare	MatrixCare	Other (Please list):	N/A: Don't have EMR
☐	☐		☐

7. To what degree is the hospital EMR "**interoperable**" (i.e., able to share information without special effort) with your facility's EMR?

	Not at all	Somewhat	Fully	N/A: Don't have EMR
a. [Hospital 1]	☐	☐	☐	☐
b. [Hospital 2]	☐	☐	☐	☐

8. Where do physicians who see patients in your facility **document their clinical notes** (select all that apply):

For patients discharged from:	(1) In hospital EHR	(2) In their own ambulatory EHR	(3) In SNF EMR	(4) Other
a. [Hospital 1]	☐	☐	☐	☐
b. [Hospital 2]	☐	☐	☐	☐

9. When patients are discharged to your facility, when are patients typically FIRST seen by:

	Same Day	Within 24 hours	Within 48 hours	>48 hours
**Physician (or equivalent)**	☐	☐	☐	☐
**NP (or equivalent)**	☐	☐	☐	☐

© 2021 Adler-Milstein J. *JAMA Network Open*.
